# New multi-criteria decision-making technique based on neutrosophic axiomatic design

**DOI:** 10.1038/s41598-022-14557-4

**Published:** 2022-06-23

**Authors:** Mohamed Abdel-Basset, Mai Mohamed, Nehal N. Mostafa, Ibrahim M. El-Henawy, Mohamed Abouhawwash

**Affiliations:** 1 Faculty of Computers and Informatics, Zagazig University, Shaibet an Nakareyah, Zagazig, 44519 Ash Sharqia Governorate Egypt; 2 Department of Mathematics, Faculty of Science, Mansoura University, Mansoura, 35516 Egypt

**Keywords:** Mathematics and computing, Engineering

## Abstract

There are several multicriteria decision-making (MCDM) approaches presented in the literature with their characteristics. Although traditional MCDM approaches are considered a proper implementation to select the best alternative from available types, they failed to consider uncertainty which is quite high and desires to be thoughtfully measured in the selection process. This research focuses on extending MCDM in the neutrosophic environment using axiomatic design (AD) as a novel contribution to selecting appropriate Computed Tomography (CT) devices. We present a new linguistic scale for evaluating criteria and alternatives based on single-valued triangular neutrosophic numbers (SVTrN). The proposed approach is superior to other existing approaches due to its simplicity and ability to simulate natural human thinking via considering truth, indeterminacy, and falsity degrees. Then, applying it will increase the value of imaging for medical decision-making and decrease needless costs. So, this study can be valuable to researchers by helping them consider the appropriate medical imaging system selection problem theoretically under uncertainty, and for governments and organizations to design better satisfying medical imaging evaluation systems.

## Introduction

Many fields are affected by DM challenges. In DM issues, it is common to study the evidence depending on many criteria instead of a single criterion. Furthermore, as the engineering environment gets more complicated, it has become more difficult for a decision-maker to assess all important parts of an issue. So, difficult decision issues are usually handled by a group of specialists by combining their expertise. Many strategies have been developed to address these difficult MCDM issues.

Axiomatic design (AD) principles are designed to provide a rigorous scientific foundation for designers, particularly in product and software design, and are frequently utilized to tackle a wide range of design challenges. It is a technique for expressing design objects as well as a set of axioms for evaluating relationships between desired functions and ways of achieving them. The use of AD principles allows for the selection of not just the best option within a set of criteria, but also the most fitting alternative. Lately, research-based on AD principles has been offered to solve MCDM challenges. The AD approach, for example, has been used to tackle problems such as cellular manufacturing systems^1^, robot arm selection^2^, and design for manufacturing context^3^. Also, Kulak et al.^4^ presented a literature review about applications of AD.

Several areas of engineering cannot be assessed quantitatively but rather qualitatively when applying AD principles to MCDM issues. this can be obtained with unclear or uncertainty information, particularly in the initial stages of engineering, the data provided is sometimes restricted and imprecise. Decisions must be made in the face of uncertain, imprecise, and ambiguous data. In this situation, it is preferable for decision makers to express their opinions using linguistic variables (LV) rather than numerical ones due to their lack of information about the issue.

As a result, fuzzy sets (FS) are introduced to deal with LV assessment. The decision of membership lies at the heart of fuzzy sets. FS used to handle uncertain information which exist usually in reality, several researchers were motivated to apply it in a fuzzy environment. These applications involved design analysis^5^, manufacturing system assessment^6^, equipment choice^7^, valuation of transportation companies^8^, seat assessment regarding ease of use aspects^9^, suggesting competitive plans on Turkish container ports^10^, the model choice for ship supervision^11^, docking execution of shipyards^12^, material choice^13,14^, training associate choosing^15^, naval design^16^, choice of sustainable energy alternative^17,18^, choosing a design concept^19^, choosing green provider^20^, and convenient choice for innovative engineering technology^21^, selection of sustainable manufacturing^22^, production research^23^, anti-vibration optimization^24^, chemistry industries^25^, and medical imaging systems^26^.

However, in many cases, the given knowledge is insufficient to precisely define the degree of membership for a certain part. An amount of hesitation can be existed between membership and non-membership. As a result of the scarcity of knowledge, intuitionistic fuzzy sets (IFSs), has been introduced as generalization of FS. IFSs, which have a membership function and a non-membership function, are more suited for coping with vagueness and ambiguity. Many researches present AD using IFS^27^. Büyüközkan et al.^28^ presented a methodology based on IFS and AD for supplier selection. The authors based on the AHP approach for defining weights of criteria. Also, Kuroshi and Ölçer^29^ presented a study on choosing and evaluating ballast water management methods via integrating IFS and AD.

The NS can overcome limitations of the fuzzy set (FS) and IFS via considering truth, intermediacy, and falsity membership degrees and its ability to distinguishe between relative truth and absolute truth, as well as among relative falsity and absolute falsity^30–32^. So, several studies motivated to present MCDM approaches in the neutrosophic environment^33–37^.

While there have been several implementations of neutrosophic MCDM approaches in the medical care domain, no implementations of NAD principles for selecting suitable CT scan devices were observed in previous studies, and this motivated us to present this study.

The key challenges behind the current study are presented as follows:

- The traditional AD principles have been extended under various uncertain environments, but all existing AD approaches have limitations in managing inconsistent and indeterminate data.
- The existing techniques employed in the literature for medical imaging system selection are not suitable for handling undefined, unknown, and unreliable information.
- Several researchers applied a limited number of quantitative or qualitative criteria with their mathematical programming models for medical imaging system selection.
- Almost existing approaches which extended AD in the fuzzy and intuitionistic fuzzy environment used a limited and inflexible linguistic scale that failed to consider indeterminacy and forced decision-makers to relate linguistic variables (LV) with fixed confirmation degrees.
- There does not exist any study in literature which presented AD in neutrosophic environment for medical image selection, therefore, it is significant to introduce a new decision‐making framework based on NAD.

The key contributions of this study are as follows:

- A novel extended MCDM methodology depending on NAD principles is established to select appropriate CT devices.
- A new linguistic scale for evaluating criteria and alternatives is presented.
- Our approach helps decision-makers to construct a logical and precise decision matrix based on a non-restrict confirmation degree with LV.
- A descriptive study of CT medical image device choosing is presented to clear the usefulness and realism of the introduced method.
- Evaluation with the present approach and sensitivity analysis are debated to explore the power and reliability of the gained decision outcomes.
- Applying the proposed approach will increase the value of imaging for medical decision-making and decrease needless costs.

The remaining sections are divided as follows: “Preliminaries” section presents preliminaries of NS and AD. “Proposed approach for medical image modalities selection” section, presents the steps of the proposed approach. “Case study: results and analysis” section presents the application of the proposed approach for medical image modalities selection. “Sensitivity analysis” section provides the sensitivity analysis. “Comparative analysis” section illustrates the comparative study. “Managerial implications” section provides the managerial implications of this study. “Conclusions and future directions” section illustrates the conclusion and future directions of this work.

## Preliminaries

In this part, some significant concepts of NS and AD principles are presented.

### Neutrosophic concepts^38^

#### Neutrosophic set (NS)

Let $\xi$ be the universe, and NS is D in $\xi$ described by a *T* function ${T}_{D}$, *I* function ${I}_{D}$ and a *F* function ${F}_{D}$ where ${T}_{D}$, ${I}_{D}$ and ${F}_{D}$ are real standard elements of [0,1]. It can be represented as:$D=\left\{<x,\left({T}_{D}\left(x\right),{I}_{D}\left(x\right),{F}_{D}\left(x\right)\right)>:x\in E,{T}_{D},{I}_{D},{F}_{D}\in \left]{0}^{-},{1}^{+}\right[\right\}$.

There is no limitation on the sum of ${T}_{D}\left(x\right),{I}_{D}\left(x\right), \; and \; {F}_{D}\left(x\right)$. So,

1 $${0}^{-}\le {T}_{D}\left(x\right)+{I}_{D}\left(x\right)+{F}_{D}\left(x\right)\ge {1}^{+}.$$

#### Score function (SF) and accuracy function (AF)

Is appropriate functions for comparing SVN. Assume $\stackrel{\sim }{{D}_{1}}=({T}_{1},{I}_{1},{F}_{1})$ be a SVN, then, the $SF(\stackrel{\sim }{{D}_{1})}$, $AF(\stackrel{\sim }{{D}_{1})}$ of a SVNN are defined as follows:

2 $$SF(\stackrel{\sim }{{D}_{1})}=(2+{T}_{1}-{I}_{1}-{F}_{1})/3$$

3 $$AF(\stackrel{\sim }{{D}_{1})}={T}_{1}-{F}_{1}$$

#### (SVTrN-number) ($\tilde{D }=<{(a}_{1},{b}_{1},{c}_{1});{T}_{D}, {I}_{D},{F}_{D}>$)

**Figure 1:**
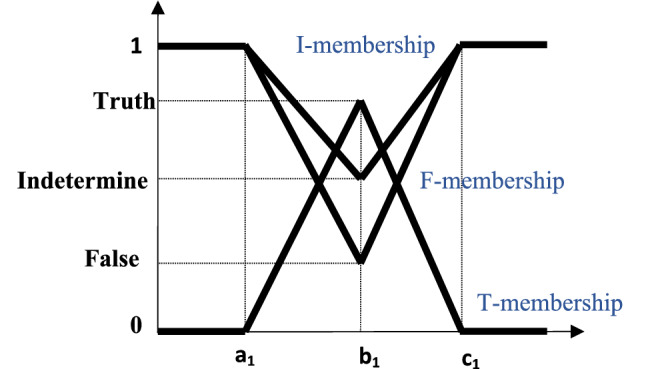
A standard single valued triangular neutrosophic number^38^.

Is a particular NS on the real number set R, whose *T, I, F* memberships are showed in Fig. 1, and represented as follows:

4 $${T}_{D}(x)=\left\{\begin{array}{c}\left(x-{a}_{1}\right){T}_{D}/\left({b}_{1}-{a}_{1}\right),\left({a}_{1}\le x<{b}_{1}\right)\\ \begin{array}{c}{T}_{D} ,({x=b}_{1})\\ \begin{array}{c}\left({c}_{1}-x\right){T}_{D}/\left({c}_{1}-{b}_{1}\right),\left({b}_{1}<x\le {c}_{1}\right)\\ 0 ,otherwise\end{array}\end{array}\end{array}\right.$$

5 $${I}_{D}(x)=\left\{\begin{array}{c}\left({b}_{1}-x-{I}_{D}(x-{a}_{1})\right)/\left({b}_{1}-{a}_{1}\right),\left({a}_{1}\le x<{b}_{1}\right)\\ \begin{array}{c}{I}_{D} ,{(x=b}_{1})\\ \begin{array}{c}\left({x-b}_{1}{+I}_{D}({c}_{1}-x)\right)/\left({c}_{1}-{b}_{1}\right),\left({b}_{1}<x\le {c}_{1}\right)\\ 1 ,otherwise\end{array}\end{array}\end{array}\right.$$

6 $${F}_{D}(x)=\left\{\begin{array}{c}\left({b}_{1}-x+{F}_{D}(x-{a}_{1})\right)/\left({b}_{1}-{a}_{1}\right),\left({a}_{1}\le x<{b}_{1}\right)\\ \begin{array}{c}{F}_{D} ,{x=b}_{1}\\ \begin{array}{c}\left(x-{c}_{1}+{F}_{D}({c}_{1}-x)\right)/\left({c}_{1}-{b}_{1}\right),\left({b}_{1}<x\le {c}_{1}\right)\\ 1 ,otherwise\end{array}\end{array}\end{array}\right.$$

#### Operations of SVTrN-number

If $\stackrel{\sim }{{D}_{1}}=<{(m}_{1},{m}_{2},{m}_{3});{T}_{1}, {I}_{1},{F}_{1}>$ and $\stackrel{\sim }{{D}_{2}}=<{(n}_{1},{n}_{2},{n}_{3});{T}_{2}, {I}_{2},{F}_{2}>$ is two SVTrN-number, then:

7 $$\stackrel{\sim }{{D}_{1}}\oplus \stackrel{\sim }{{D}_{2}}=<{(m}_{1}+{n}_{1},{m}_{2}+{n}_{2},{m}_{3}+{n}_{3});{\mathrm{min}(T}_{1},{T}_{2}),max( {I}_{1},{I}_{2}),{\mathrm{max}(F}_{1},{F}_{2})>$$

8 $$\stackrel{\sim }{{D}_{1}}\otimes \stackrel{\sim }{{D}_{2}}=<{(m}_{1}{n}_{1},{m}_{2}{n}_{2},{m}_{3}{n}_{3});{min(T}_{1},{T}_{2}),max( {I}_{1},{I}_{2}),{max(F}_{1},{F}_{2})>$$

9 $$\lambda \stackrel{\sim }{{D}_{1}}=<{(\lambda m}_{1},\lambda {m}_{2},\lambda {m}_{3});{min(T}_{1},{T}_{2}),max( {I}_{1},{I}_{2}),{max(F}_{1},{F}_{2})>$$

#### SC and AF of SVTrN-number

The SF $s(\stackrel{\sim }{{D}_{1})}$ and AF $a(\stackrel{\sim }{{D}_{1})}$ can be defined as follows:

10 $$SF(\stackrel{\sim }{{D}_{1})}=\left(\frac{1}{12}\right)[{(m}_{1}+2{m}_{2}+{m}_{3} ]\times [2+{T}_{1}-{I}_{1}-{F}_{1}]$$

11 $$AF(\stackrel{\sim }{{D}_{1})}=\left(\frac{1}{12}\right)[{(m}_{1}+2{m}_{2}+{m}_{3} ]\times [2+{T}_{1}-{I}_{1}+{F}_{1}]$$

#### Ranking of SVTrN-number

12 $$\mathrm{If} \; SF(\stackrel{\sim }{{D}_{1})}< SF(\stackrel{\sim }{{D}_{2})}, then \stackrel{\sim }{{D}_{1}}<\stackrel{\sim }{{D}_{2}}$$

13 $$\mathrm{If}  \; SF(\stackrel{\sim }{{D}_{1})}= SF(\stackrel{\sim }{{D}_{2})}, and \,if$$

$$AF(\stackrel{\sim }{{D}_{1})}< AF(\stackrel{\sim }{{D}_{2})},  \; then  \; \stackrel{\sim }{{D}_{1}}<\stackrel{\sim }{{D}_{2}}$$

$$AF(\stackrel{\sim }{{D}_{1})}> AF(\stackrel{\sim }{{D}_{2})},  \; then  \; \stackrel{\sim }{{D}_{1}}>\stackrel{\sim }{{D}_{2}}$$

$$AF(\stackrel{\sim }{{D}_{1})}= AF(\stackrel{\sim }{{D}_{2})}, \; then \; \stackrel{\sim }{{D}_{1}}=\stackrel{\sim }{{D}_{2}}$$

### Axiomatic design principles

Independence axiom and information axiom are the most significant concepts of AD principles. The independence of functional requirements (FRs) that must be implemented is stated by the independence axiom. FRs indicate the smallest set of independent requirements which exemplifies the design goals. Where one of the most significant advantages of these methods is that if an alternative does not fulfil the FRs, the model stops it from being chosen as the best option.The information axiom declares that the design which has the minimum information content (IC) is the finest design between the designs that meet the independence axiom^39^.

The information axiom is represented by the IC which is correlated to the probability of sustaining the plan goals. The ${IC}_{i}$ is given by:

14 $${IC}_{i}={\mathrm{log}}_{2} \left(\frac{1}{{\mathrm{Probability}}_{i}}\right),$$

since ${\mathrm{Probability}}_{i}$ is the probability of reaching a certain function requirement. . The logarithmic function is selected so that the IC will be additive when there are several FRs that must be fulfilled simultaneously. Where there are n FRs, the total IC is the total of all these probabilities^39^.

**Figure 2:**
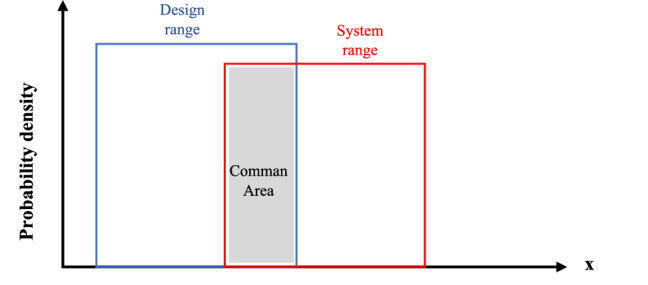
The common area of SR and DR^26^.

The probability of accomplishment is specified by the designer needs to attain regard to design range (DR) and the needs capability of the system regard to system range (SR). The intersection area of the DR and the SR is the common area where the satisfactory solution exists, as appears in Fig. 2.

In the case of the uniform probability distribution function ${\mathrm{Probability}}_{i}$ can be presented as follows:

15 $${\mathrm{Probability}}_{i}=(Common \; range/System\;  range),$$

16 $${IC}_{i}={\mathrm{log}}_{2}(System\; range/Common \;range).$$

## Proposed approach for medical image modalities selection

In the current section, the important concepts of AD in a neutrosophic environment are introduced. Also, a new MCDM approach for selecting appropriate medical image modalities is presented.

### The extend of AD principles in neutrosophic environment

**Figure 3:**
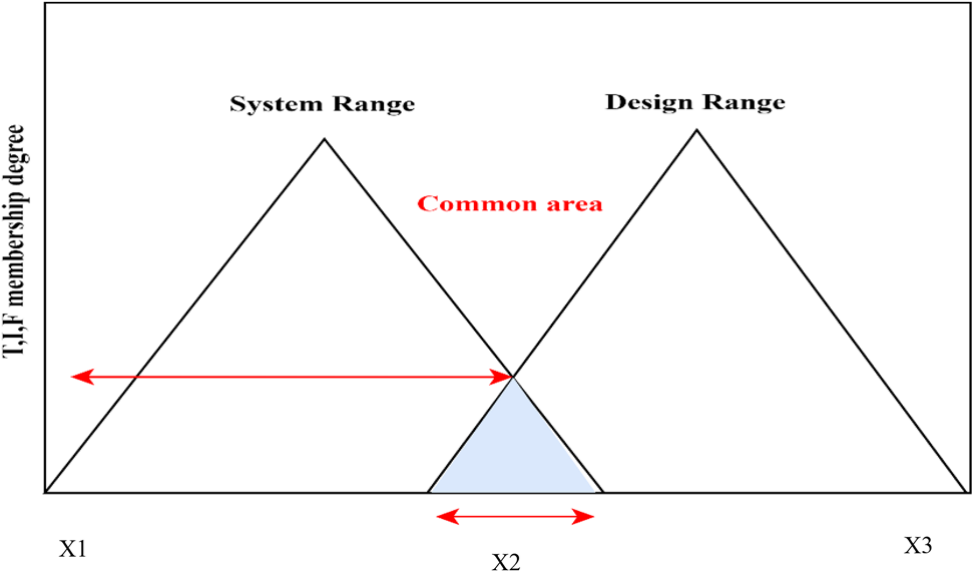
The intersection region between SR and DR of neutrosophic numbers.

The values of criteria are presented using LV under the neutrosophic domain. Since we have imperfect information about SR and DR, then SR and DR for a specific criterion will be stated by utilizing “truth-membership (TM),” “falsity-membership (FM)” and “indeterminacy-membership (IM).” Therefore, the intersection areas of TM, IM, and FM functions of neutrosophic numbers can be attained as demonstrated in Fig. 3.

The TM, IM, and FM of information content are represented as $$({IC}^{T})$$, ($${IC}^{I})$$, and $$({IC}^{F})$$ respectively which can be expressed as follows:

17 $${IC}^{T}={\mathrm{log}}_{2}\frac{\mathrm{Truth-}\mathrm{membership \; system \; desgin}}{\mathrm{Truth-}\mathrm{membership \; Common \;area}}$$

18 $${IC}^{I}={\mathrm{log}}_{2}\frac{\mathrm{Indeterminacy-}\mathrm{membership \; system \; desgin}}{\mathrm{Indeterminacy-}\mathrm{membership \;Common \;area}}$$

19 $${IC}^{F}={\mathrm{log}}_{2}\frac{\mathrm{Falsity-}\mathrm{membership \; system \; desgin}}{\mathrm{Falsity-}\mathrm{membership \; Common \; area}}$$

In NS-domain, the SF and the AF are used to compare neutrosophic values that can be expressed as $s$ and $a$ respectively. In this approach, we expand $s$ and $a$ with the IC in the AD environment. We represent ${SF}_{i}$ and ${AF}_{i}$ based on IC as represented in Eqs. (2) and (3).

### The approach for MCDM problems based on AD principles and neutrosophic environment

If $AT=\left\{{AT}_{1},{AT}_{2},\ldots,{AT}_{m}\right\}$ is a group of alternatives, $C=\left\{{C}_{1},{C}_{2},\ldots,{C}_{n}\right\}$ is a group of criteria, $we=\left\{{We}_{1},{We}_{2},\ldots,{We}_{n}\right\}$ is the weight for each $C$, where ${We}_{j}\ge 0$, $j=\mathrm{1,2},\dots ,n$ ,$\sum_{j=1}^{n}{We}_{j}=1$, $DM=\left\{{DM}_{1},{DM}_{2},\ldots,{DM}_{t}\right\}$ is a group of DM, with an important degree of ${D}_{i}$ is ${\eta }_{i}\in [\mathrm{0,1}]$ and $\sum_{v=1}^{t}{\eta }_{v}=1$ and $FR=\left\{{\widehat{f}}_{r1},{\widehat{f}}_{r2},\ldots,{\widehat{f}}_{rn}\right\}$ is a group of FRs, that is the group of objectives for the criteria, $\widehat{{g}_{j}}=(<{\widehat{g}}_{j1},{\widehat{g}}_{j2},{\widehat{g}}_{j3}>{a}_{j}, {\theta }_{j},{\beta }_{j})\in FR$ based on SVTrN-number. If $\widehat{{P}_{k}}$ is a set of DM matrices where $\widehat{{P}_{ij}^{(k)}}=(<{p}_{ij1}^{(k)},{p}_{ij2}^{(k)},{p}_{ij3}^{(k)}>{a}_{ij}^{(k)},{\theta }_{ij}^{(k)},{\beta }_{ij}^{(k)})\in$ $\widehat{{P}_{k}}$ is a preference value that is represented as SVTrN-number by the decision-maker, ${DM}_{K}\in DM$ for ${AT}_{i}\in AT$ regarding to ${C}_{j}\in C$.

The proposed methodology of MCDM problems based on NAD for medical image modality selection are as follows:

Step 1.: Represent data using SVTrN which has the form of $<(\mathrm{Lower \; value}(\mathrm{L}),\mathrm{Medium }(\mathrm{M}),\mathrm{Upper  \;value}(\mathrm{U}));\mathrm{ confirmation  \; degree }(\mathrm{CD})>$. Use Table 1 for representation process of data.

**Table 1:** Linguistic variables of criteria and alternatives in the form of SVTrN-number.

Terms	$L, M, U$	Confirmation degree ($T,I,F$)
Absolutely low (AL)	$< (\mathrm{0,0},1)>$	Absolutely not sure (ANS)$<(\mathrm{0,1},1)>$
Very low (VL)	$< (\mathrm{0,1},2)>$	Not sure (NS)$<(\mathrm{0.2,0.8,0.8})>$
Low (L)	$< (\mathrm{1,2},3)>$	Slightly sure (SLS)$<(\mathrm{0.3,0.7,0.7})>$
Medium (M)	$< (\mathrm{2,3},4)>$	Median sure (MS)$<(\mathrm{0.5,0.5,0.5})>$
High (H)	$< (\mathrm{3,4},5)>$	Sure(S)$<(\mathrm{0.7,0.4,0.4})>$
Very high (VH)	$< (\mathrm{4,5},6)>$	Strongly sure (STS)$<(\mathrm{0.8,0.2,0.2})>$
Strongly very high (SVH)	$< (\mathrm{5,6},7)>$	Very strongly sure (VSS)$<(\mathrm{0.9,0.1,0.1})>$
Absolutely high (AH)	$< (\mathrm{7,8},9)>$	Absolutely sure (AS)$< (\mathrm{1,0},0)>$
Step 2.: Aggregate decision-makers opinions using the average method via using Eq. (7) to get the summation of SVTrN-numbers and then divide it on their numbers for each criterion.
Step 3.: Compute the ${IC}^{T}$ for each ${FR}_{i}$.
  20 $${IC}_{ij}^{T}=\left\{ {\begin{array}{ll}    0 & \quad {if\;\widehat{p}_{{ij1}}  > \widehat{g}_{{j3}} \;or\;\widehat{p}_{{ij3}}  > \widehat{g}_{{j1}} }  \\    {\log _{2} \frac{{Truth{\text{-}}membership\;system\;design}}{{{\text{Truth-}} membership\;{\text{common}}\;{\text{area}}}}} & \quad {if\;\widehat{p}_{{ij1}}  \le \widehat{g}_{{j3}} \;or\;\widehat{p}_{{ij3}}  \le \widehat{g}_{{j1}} }  \\   \end{array} } \right. $$
  where ${\widehat{p}}_{ij1}$ and ${\widehat{p}}_{ij3}$ are the $L$ and $U$ values of ${AT}_{i}$ by ${C}_{i}$, where ${\widehat{g}}_{j1}$ and ${\widehat{g}}_{j3}$ are *L* and *U* values of ${FR}_{i}$.
Step 4.: Compute the ${IC}^{I}$ for each ${FR}_{i}$.
  21 $${IC}_{ij}^{I}=\left\{ {\begin{array}{ll}    0 & \quad if \; {\widehat{p}}_{ij1} > {\widehat{g}}_{j3} \; or \; {\widehat{p}}_{ij3} > {\widehat{g}}_{j1}  \\    {\log _{2} \frac{{{\text{Indeterminacy-}}membership\;system\;design}}{{{\text{Indeterminacy-}} membership\;{\text{common}}\;{\text{area}}}}} & \quad {if\;\widehat{p}_{{ij1}}  \le \widehat{g}_{{j3}} \;or\;\widehat{p}_{{ij3}}  \le \widehat{g}_{{j1}} }  \\   \end{array} } \right.$$
  where ${\widehat{p}}_{ij1}$ and ${\widehat{p}}_{ij3}$ are the *L* and *U* values of ${AT}_{i}$ by ${C}_{i}$, where ${\widehat{g}}_{j1}$ and ${\widehat{g}}_{j3}$ are *L* and *U* values of ${FR}_{i}$.
Step 5.: Compute the ${IC}^{F}$ for each ${FR}_{i}$.
  22 $${IC}_{ij}^{F}=\left\{ {\begin{array}{ll}    0 & \quad if \; {\widehat{p}}_{ij1} > {\widehat{g}}_{j3} \; or \; {\widehat{p}}_{ij3} > {\widehat{g}}_{j1}  \\    {\log _{2} \frac{{{\text{False-}}membership\;system\;design}}{{{\text{False-}} membership\;{\text{common}}\;{\text{area}}}}} & \quad {if\;\widehat{p}_{{ij1}}  \le \widehat{g}_{{j3}} \;or\;\widehat{p}_{{ij3}}  \le \widehat{g}_{{j1}} }  \\   \end{array} } \right.$$
  where ${\widehat{p}}_{ij1}$ and ${\widehat{p}}_{ij3}$ are the *L* and *U* values of ${AT}_{i}$ by ${C}_{i}$, where ${\widehat{g}}_{j1}$ and ${\widehat{g}}_{j3}$ are *L* and *U* values of ${FR}_{i}$.
Step 6.: Compute the value of SF for IC of ${AT}_{i}$
  After computing the ${IC}_{ij}^{T},{IC}_{ij}^{I}$, and ${IC}_{ij}^{F}$ we get the form of SVN. So SF is computed based on Eq. (2).
  23 $${SF}_{i}=\sum_{j=1}^{n}{SF}_{ij}\cdot{W}_{j}=\sum_{j=1}^{n}[2+{T}_{ij}-{I}_{ij}-{F}_{ij}]/3\cdot{W}_{j}$$
Step 7.: Compute the value of AF for IC of ${AT}_{i}$.
  After computing the ${IC}_{ij}^{T},{IC}_{ij}^{I}$, and ${IC}_{ij}^{F}$ we get the form of SVN. So AF is computed based on Eq. (3).
  24 $${AF}_{i}=\sum_{j=1}^{n}{AF}_{ij}\cdot{W}_{j}=\sum_{j=1}^{n}[{T}_{ij}-{F}_{ij}]\cdot{W}_{j}$$
Step 8.: Rank alternatives.
  Based on ranking of SVTrN-number using Eqs. (12) and (13), choose the best alternative, according to ${SF}_{i}$ . In the condition that ${SF}_{i}$ of alternatives are equal, then rank them based on ${AT}_{i}$. This part presented as follows:
  25 $$\mathrm{If} \; {SF}_{i}<{SF}_{j}, \; then\, {AT}_{i}<{AT}_{j} \; (i.e. {AT}_{i} \; is \; worse \; than \; {AT}_{j})$$
  26 $${\mathrm{If }} \;\;SF_{i}={SF}_{j} \;  and \;  if,$$
  $${AF}_{i}< {AF}_{j}, \;  then \;  {AT}_{i}<{AT}_{j} \;  (i.e. {AT}_{i} \;  is \;  worse \;  than \; {AT}_{j})$$
  $${AF}_{i}> {AF}_{j}, \;  then \;  {AT}_{i}>{AT}_{j} \; (i.e. {AT}_{i} \;  is \;  better \;  than \;  {AT}_{j} )$$
  $${AF}_{i}={AF}_{j}, \;  then \;  {AT}_{i}={AT}_{j} \;  (i.e. {AT}_{i} \;  is \;  equal \;  to \;  {AT}_{j})$$

**Figure 4:**
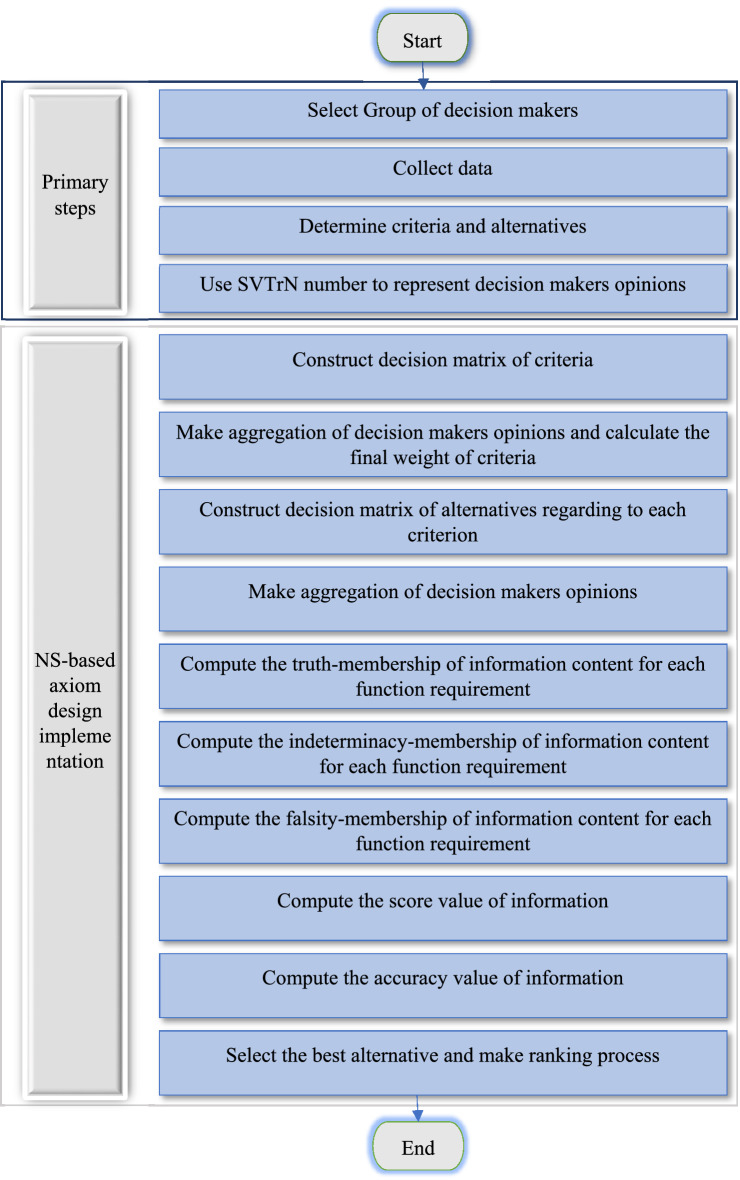
Flowchart of proposed approach.

The flowchart of proposed approach presented in Fig. 4.

## Case study: results and analysis

In our study, we aim to introduce a novel methodology for selecting the suitable medical image modality for a hospital involve 3000 employees. The selection process is based on five available devices of CT medical image modalities. The decision-makers in our case study are as follows:

- D1: The main physician,
- D2: A purchasing sector director, and
- D3: A radiologist.

The decision-makers detected four criteria to select the available device as follows:

- C1: Number of image slices, C2: Price, C3: Highest patient weight (kg), and C4: Excellence of After-sales service. Also, the FRs that must be assured by the CT device are presented in Table 2.

**Table 2:** FRs for CT modality.

Criteria	Functional requirements	Neutrosophic value
C1	VH; STS	$< (\mathrm{4,5},6) ;(\mathrm{0.8,0.2,0.2})>$
C2	M; STS	$< (\mathrm{2,3},4) ;( \mathrm{0.8,0.2,0.2})>$
C3	SVH; VSS	$< (\mathrm{5,6},7) ;(\mathrm{0.9,0.1,0.1})>$
C4	AH; VSS	$< (\mathrm{7,8},9) ;(\mathrm{0.9,0.1,0.1})>$

For solving this MCDM problem using the suggested approach apply the following steps:

Step 1.: Use LV presented in Table 1 to represent data. After then, construct decision matrices according to decision makers' selections for determining the significance rate of criteria as in Table 3.

**Table 3:** The neutrosophic values for evaluating criteria regarding decision-makers.

	C1	C2	C3	C4
D1	AH; AS	L; NS	AL; NS	M; ANS
D2	H; MS	M; NS	H; MS	H; VSS
D3	M; STS	VL; S	M; SLS	L; MS

The corresponding SVTrN values				
	C1	C2	C3	C4
D1	$(\mathrm{7,8},9) ;(\mathrm{1,0},0)$	$(\mathrm{1,2},3) ;(\mathrm{0.2,0.8,0.8})$	$(\mathrm{0,0},1) ;(\mathrm{0.2,0.8,0.8})$	$(\mathrm{2,3},4) ;(\mathrm{0,1},1)$
D2	$(\mathrm{3,4},5) ;(\mathrm{0.5,0.5,0.5})$	$(\mathrm{2,3},4) ;(\mathrm{0.2,0.8,0.8})$	$(\mathrm{3,4},5) ;(\mathrm{0.5,0.5,0.5})$	$(\mathrm{3,4},5) ;(\mathrm{0.9,0.1,0.1})$
D3	$(\mathrm{2,3},4) ;(\mathrm{0.8,0.2,0.2})$	$(\mathrm{0,1},2) ;(\mathrm{0.7,0.4,0.4})$	$(\mathrm{2,3},4) ;(\mathrm{0.3,0.7,0.7})$	$(\mathrm{1,2},3) ;(\mathrm{0.5,0.5,0.5})$
Step 2.: Use the average method for aggregating weights of decision makers and make normalization after that as in Table 4. Finally, use the SF equation to compute weights values.

**Table 4:** The aggregated and normalized values of weights.

	C1	C2	C3	C4
Aggregated weights	$(\mathrm{4,5},6) ;(\mathrm{0.5,0.5,0.5})$	$(\mathrm{1,2},3) ;(\mathrm{0.2,0.8,0.8})$	$(\mathrm{1.67,2.3,3.3}) ;(\mathrm{0.2,0.8,0.8})$	$(\mathrm{2,3},4) ;(\mathrm{0,0.5,0.5})$
Normalized weights	$(\mathrm{1,1},1) ;(\mathrm{0.5,0.5,0.5})$	$(\mathrm{0.25,0.4,0.5}) ;(\mathrm{0.2,0.8,0.8})$	$(\mathrm{0.4175,0.46,0.55}) ;(\mathrm{0.2,0.8,0.8})$	$(\mathrm{0.5,0.6,0.67}) ;(\mathrm{0,0.5,0.5})$
SF for weights	$0.5$	$0.0775$	$0.094375$	$0.1975$
Step 3.: Construct decision matrices for evaluating alternatives considering every criterion as in Table 5.

**Table 5:** Evaluating alternatives considering every criterion.

D1				
	C1	C2	C3	C4
A1	H; S	M; MS	AH; VSS	AH; STS
A2	SVH; STS	M; S	SVH; VSS	AH; STS
A3	M; MS	L; SLS	AH; VSS	AH; VSS
A4	AH; VSS	L; MS	M; MS	AH; VSS
A5	AH; VSS	VL; VSS	H; S	AH; VSS

D2				
	C1	C2	C3	C4
A1	VH; S	M; MS	VH; STS	SVH; VSS
A2	VH; STS	L; SLS	VH; VSS	SVH; VSS
A3	M; MS	VL; SLS	H; S	SVH; STS
A4	AH; VSS	L; SLS	VH; STS	VH; STS
A5	H; S	VL; NS	H; S	AH; VSS

D3				
	C1	C2	C3	C4
A1	VH; VSS	M; MS	H; S	AH; VSS
A2	H; S	VL; NS	H; S	SVH; STS
A3	VH; VSS	L; SLS	H; MS	VH; STS
A4	H; STS	VL; SLS	AH; STS	SVH; STS
A5	H; S	H; MS	SVH; STS	AH; VSS
Step 4.: Make aggregation of decision makers' opinions about evaluating alternatives considering every criterion as in Table 6.

**Table 6:** The aggregated values of alternatives.

	**C1**	C2	C3	C4
A1	$< \left(\mathrm{3.67,4.67,5.67}\right);(\mathrm{0.7,0.4,0.4}) >$	$< \left(\mathrm{2,3},4\right);(\mathrm{0.5,0.5,0.5})>$	$< \left(4.67, \mathrm{5.67,6.67}\right);(\mathrm{0.7,0.4,0.4})>$	$< \left(\mathrm{6.33,7.33,8.33}\right);(\mathrm{0.8,0.2,0.2})>$
A2	$< \left(\mathrm{4,5},6\right);(\mathrm{0.7,0.4,0.4})>$	$< \left(\mathrm{1,2},3\right);(\mathrm{0.2,0.8,0.8})>$	$< \left(\mathrm{4,5},6\right);(\mathrm{0.7,0.4,0.4})>$	$< \left(\mathrm{5.67,6.67,7.67}\right);(\mathrm{0.8,0.2,0.2})>$
A3	$< \left(2.67, \mathrm{3.67,4.67}\right);(\mathrm{0.5,0.5,0.5})>$	$< \left(0.67, 1.67, 2.67\right);(\mathrm{0.3,0.7,0.7})>$	$< \left(4.33, 5.33, 6.33\right);(\mathrm{0.5,0.5,0.5})>$	$< \left(\mathrm{5.33,6.33,7.33}\right);(\mathrm{0.8,0.2,0.2})>$
A4	$< \left(5.67, \mathrm{6.67,7.67}\right);(\mathrm{0.80,0.20}, 0.20)>$	$< \left(\mathrm{0.67,1.67,2.67}\right);(\mathrm{0.3,0.7,0.7})>$	$< \left(\mathrm{4.33,5.33,6.33}\right);(\mathrm{0.5,0.5,0.5})>$	$< \left(\mathrm{5.33,6.33,7.33}\right);(\mathrm{0.8,0.2,0.2})>$
A5	$< \left(4.33, \mathrm{5.33,6.33}\right);(\mathrm{0.7,0.4,0.4})>$	$< \left(\mathrm{1,2},3\right);(\mathrm{0.2,0.8,0.8})>$	$< \left(3.67, 4.67, 5.67\right);(0.7, \mathrm{0.4,0.4})>$	$< \left(\mathrm{7,8},9\right);(\mathrm{0.9,0.1,0.1})>$
Step 5.: Compute the ${IC}^{T}$ using Eq. (20) as presented in Table 7.

**Table 7:** The Truth-membership IC.

	C1	C2	C3	C4
A1	$0.497$	0	0.497	$1.162$
A2	$0$	$1.23$	$1.807$	$3.415$
A3	$3.05$	$2.836$	$0.873$	$5.473$
A4	$4.64$	$2.836$	$0.873$	$5.473$
A5	$0.341$	$1.23$	$3.22$	$0$
Step 6.: Compute the ${IC}^{I}$ using Eq. (21) as presented in Table 8.

**Table 8:** The Indeterminacy-membership IC.

	C1	C2	C3	C4
A1	$1.3$	$1.32$	$2.32$	$1.7$
A2	$1$	$3.32$	$3.152$	$4.058$
A3	$4.21$	$4.544$	$2.943$	$6.058$
A4	$4.32$	$4.544$	$2.943$	$6.058$
A5	$1.234$	$3.32$	$1.736$	$0$
Step 7.: Compute the ${IC}^{F}$ using Eq. (22) as presented in Table 9.

**Table 9:** The False-membership IC.

	C1	C2	C3	C4
A1	$1.3$	$1.32$	$2.32$	$1.7$
A2	$1$	$3.32$	$3.152$	$4.058$
A3	$4.21$	$4.544$	$2.943$	$6.058$
A4	$4.32$	$4.544$	$2.943$	$6.058$
A5	$1.234$	$3.32$	$1.736$	$0$
Step 8.: Compute the SF of IC using Eq. (23) as appears in Table 10.

**Table 10:** SF results.

A1	A2	A3	A4	A5
$-0.13$	$-0.433366875$	$-1.224790208$	$-0.9964569$	$0.143230833$
Step 9.: Compute the AF of IC using Eq. (24) as appears in Table 11.

**Table 11:** AF results.

A1	A2	A3	A4	A5
$-0.85$	$-0.979398125$	$-1.0810325$	$-0.3410325$	$-0.4684225$
Step 10.: Ranking alternatives
  Based on Eqs. (25) and (26) for ranking NS numbers. The final rank is as follows: $A5>A1>A2>A4>A3$.

## Sensitivity analysis

The sensitivity analysis of the suggested approach is conducted to assess the persistence of the priority rating and it can be an efficient way to determine the proposed approach’s efficiency. A sensitivity analysis was performed on the attribute rank. So, we will show how various priorities of criteria will impact on final rank of alternatives.

**Figure 5:**
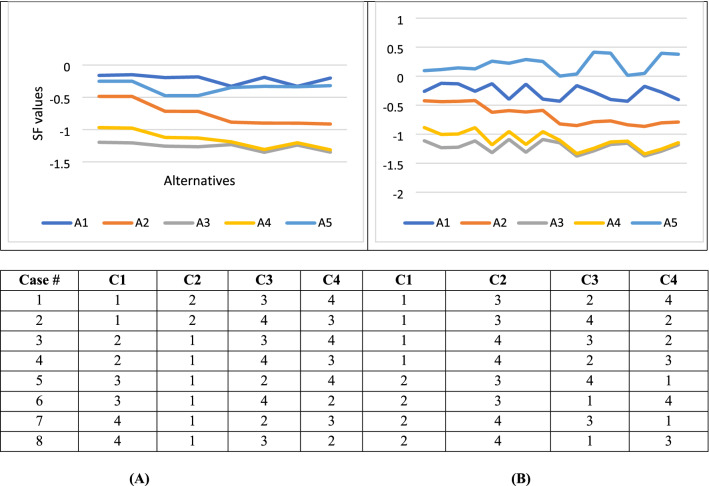
Various priorities of criteria and its impact on alternatives rank from case 1 to 8.

As we have 5 attributes, we get 24 case, so only 8 random cases has been shown in Fig. 5. Figure 5 show the change in the final rank of alternatives regarding to various priorities of criteria. As shown in the figure, changing the order of the attributes has a significant affect on the weights of the alternatives.

The result of our sensitivity analysis shows that alternative1 and 5 are the best two alternatives for CT device selection. Alternative 1 shows superior results in Fig. 5A, while alternative 5 shows superior ranking results in Fig. 5B. Alternatives 4 and 3 are the lowest rank of alternatives in all cases. While alternative 2 shows a medium rank in all results which indicates the stability during changes of weights.

The findings of the sensitivity analysis indicate that in cases 1, 2, 7,8,13,14, 19 and 20, A1 is the best alternative, and A3 is the worst one. In cases 3, 4, 5, 6, 9, 10, 11, 12, 15, 16, 17, 18, 21, 22, 23, and 24, A5 is the best CT device and A3 is the worst. In conclusion, A1 showed eight times as the best alternative. Also, A1 showed sixteen times as the best alternative. But, A3 is the lowest rank in all cases.

## Comparative analysis

In this part, we provide a comparison between the suggested approach and the other approach which presented AD in the intuitionistic fuzzy environment^27^.

By applying the proposed approach in^27^ on our CT device selection problem, the final rank of alternatives are as follows: $A1 > A2> A5> A4>A3$. This rank is determine based on SF and AF values as in Eqs. (25) and (26). Where the highest SF value in our proposed is A5 and the highest SF value in^27^ is A1. Table 12 summarizes the final rank of our CT selection problem using the approach presented in^27^ and our suggested approach.

**Table 12:** Ranking of alternatives using two approaches.

	Proposed approach	Presented in^27^
A1	2	1
A2	3	2
A3	5	5
A4	4	4
A5	1	3

For comparing ranks of the proposed approach with other approaches presented in^27^ we used the following statistical methods as follows:

### Spearman's correlation

Calculates the linear correlation between two continuous variables. A correlation is linear when a change in one variable is correlated with a relative change in the other variable, that presented as follows:

27 $$SP=1-\left[\frac{6\cdot\sum_{n=1}^{ALT}{({Distance}_{n})}^{2}}{ALT\cdot({ALT}^{2}-1)}\right]$$

where ALT is the number of alternatives, and ${Distance}_{n}$ is the subtraction between alternatives ranking. The values of $+1$ or $-1$ indicate a strong correlation between two observations, and the $0$ value indicates a low correlation.

### Person's correlation

Calculate the strength of linear correlation. The values of $+1$ or $-1$ indicate a completely positive or negative linear correlation. and $0$ value indicates unavailable linear correlation. Which represented as follows:

28 $$PR\left(x,y\right)=\frac{cov(x,y)}{{\sigma }_{x}{\sigma }_{y}}$$

where, $cov(x,y)$ is the covariance between $x,y$, and ${\sigma }_{x},{\sigma }_{y}$ is the standard deviation of $x$ and $y$ respectively.

By calculating the Spearman correlation using Eq. (27) the correlation value is 0.7. Also, the Person correlation is 0.7, which indicates a strong correlation between the two approaches.

From a comparative study between our proposed approach and other presented approach in^27^, we concluded that our approach is simple to implement and more logical than the presented approach in^27^ for the following reasons:

- Since NS is more effective than IFS for dealing with uncertainty, then extending AD in a neutrosophic environment is more precise than AD which is presented in an intuitionistic fuzzy environment.
- The extended AD in a neutrosophic environment simulates natural human thinking since indeterminacy degree does not depend on truth and falsity degree. Then, it can deal with situations in which fuzzy and intuitionistic fuzzy AD fails to handle.
- Our proposed scale can deal with bigger areas in common ranges than presented in^27^.
- Finally, the scale presented in^27^, can’t provide a logical confirmation degree since it is restricted to the linguistic variable. But our scale makes decision-makers feel free to use the suitable linguistic variable and its confirmation degree which can vary from one decision-maker to another.

## Managerial implications

All existing hospitals need to select appropriate types of medical imaging systems which able to notice diseases at their beginning phase and then refining the patient’s prediction intensely. As the selection process is a hard and complex task due to conflicting criteria and numerous available alternatives which exist nowadays, then we need a new extended MCDM approach. In this study, we presented for the first time a new extended MCDM approach based on NAD for handling uncertainty which exist usually in the selection process. The proposed approach proved its ability to deal with uncertainty and then make precise decisions. The proposed model can be a powerful guide for hospitals or medical organizations that desire to select appropriate medical imaging systems. Also, governments can use the suggested approach for making precise decisions about any social, economic, and environmental problems.

## Conclusions and future directions

Lately, AD methods to decision making have grown in popularity. One of the most significant advantages of these methods is that if an alternative does not fulfil the FRs, the model stops it from being chosen as the best option. NAD techniques may handle both neutrosophic and crisp values simultaneously by extending the AD methodology to neutrosophic settings. This feature is not present in any other MCDM techniques described in the literature. In our study, we presented for the first time the principles of AD in a neutrosophic environment. We also suggested a new MCDM approach depending on neutrosophic axiomatic design for medical imaging systems. A real case study for selecting the best CT device is provided. To our knowledge, this study is the first in which AD technique are integrated with SVTrN.

We also compared the proposed approach with another approach that applied AD in an intuitionistic fuzzy environment. From the comparison, we concluded that our approach is superior to other approaches due to its simplicity and ability to represent natural human thinking via considering vague, and uncertain information.

In the future, we plan to use various multicriteria decision-making approaches based on neutrosophic axiomatic design to solve various problems in the engineering and agriculture domains. We also plan to implement AD to various types of NS for image modality selection.

## Data Availability

All data generated or analyzed during this study are included in this article.
